# Timing of Switching to Steroid Implants in Cases of Recalcitrant Diabetic Macular Edema Not Responding to Anti-vascular Endothelial Growth Factor (VEGF) Therapy: A Real-World Study

**DOI:** 10.7759/cureus.62385

**Published:** 2024-06-14

**Authors:** Seemant Raizada, Jamal Al Kandari, Fahad AL Diab, Khalid Al Sabah, Niranjan Kumar, Sebastian Mathew, Yousef AL Dafiri, Talal Abdul Jaleel, Mahmoud Alrabiah, Mohammad Al Ajmi

**Affiliations:** 1 Retina Unit, Kuwait Specialized Eye Center, Shaab Al Bahiri, KWT; 2 Retina Unit, Al Bahar Eye Center, Kuwait City, KWT; 3 Retina Unit, Sheikh Jaber Al Ahmad Al Sabah Hospital, Kuwait City, KWT; 4 Ophthalmology, Faculty of Medicine, Kuwait University, Kuwait City, KWT; 5 Ophthalmology, Al Bahar Eye Center, Kuwait City, KWT; 6 Ophthalmology, Kuwait Oil Company Hospital, Kuwait City, KWT

**Keywords:** intravitreal dexamethasone implants, anti-vegf, medical treatment, dme, diabetic macular edema

## Abstract

Purpose

The purpose of this study is to examine the impact of the timing of the steroid switch on both visual and anatomical outcomes in diabetic macular edema (DME) eyes that have shown an inadequate response to multiple intravitreal anti-vascular endothelial growth factor (anti-VEGF) injections. In the treatment of DME, anti-VEGF injections are typically the initial course of action. However, in cases where DME persists despite anti-VEGF treatment, intravitreal dexamethasone implants (Ozurdex^®^, Allergan Inc., Irvine, CA) are often utilized. Despite this, there remains a lack of consensus regarding the optimal timing for transitioning to steroid treatment. This study aims to shed light on the potential benefits of adjusting the timing of the steroid switch in cases of recalcitrant DME.

Methods

The eyes (n = 105) of 77 patients with recalcitrant DME were included in this retrospective, interventional, comparative study comprising three groups: participants switched to steroid implants after three anti-VEGF injections (Group I), four to six anti-VEGF injections (Group II), and more than six anti-VEGF injections (Group III). Anti-VEGF treatment failure was defined as a central retinal thickness (CRT) of ≥300 microns and/or a lack of visual improvement (≤1 line of visual gain according to Snellen acuity). The last follow-up took place after 10-12 weeks of Ozurdex^®^ injections.

Results

Improvement was observed in 19 eyes (46%), 17 eyes (50%), and 10 eyes (33%) in Groups I, II, and III, respectively, after switching to dexamethasone implants. The best overall results (an improvement in vision and stabilization) were seen in Group II (32 eyes, 94%). The decrease in CRT was statistically significant in all three groups.

Conclusion

Intravitreal dexamethasone implants improved functional and morphological outcomes in anti-VEGF-resistant DME eyes. After four to six anti-VEGF injections, switching to a steroid implant resulted in the best functional results.

## Introduction

Diabetic retinopathy (DR) is an important major microvascular complication of diabetes mellitus (DM) and the leading cause of visual loss [1]. The most frequent cause of visual impairment in DR is diabetic macular edema (DME), which occurs due to interstitial fluid and lipid accumulation and hemorrhage in the macular area [2]. The pathophysiology of DME is complex and involves vascular endothelial growth factor (VEGF) and other inflammatory mediators. Intravitreal pharmacotherapy has revolutionized the therapeutic options for DME, and visual and morphological improvements have been achieved with the treatment of intravitreal anti-VEGF agents [3,4]. Nevertheless, the efficacy of intravitreal agents is not predictable in all patients.

Data from the Diabetic Retinopathy Clinical Research Network (DRCR.net, Protocol I) were analyzed to determine the frequency of persistent DME and whether chronic persistent DME had deleterious effects on visual acuity outcomes after three years. The analysis revealed that 40% of eyes receiving monthly ranibizumab experienced persistent DME for 24 weeks. With continued treatment of the eyes according to a protocol based on changes in visual acuity and optical coherence tomography, the percentage of patients with chronic persistent DME (i.e., not resolving after two consecutive visits) was established to be 56% and 40% at the two- and three-year follow-ups, respectively [5].

In cases of clinically significant macular edema resistant to anti-VEGF therapy, retinal physicians seek alternate treatment modalities, such as intravitreal steroids. Corticosteroids assist in multiple ways in the treatment of DME since they are potent anti-inflammatory agents and inhibit VEGF expression [6-8]. The use of a dexamethasone implant (Ozurdex®; Allergan, Irvine, CA) is an alternative therapeutic approach that has been approved by the US Food and Drug Administration for DM [9-11]. Several studies have shown that in eyes with an insufficient response to anti-VEGF inhibitors, switching to dexamethasone implant injections provided better functional and morphological outcomes [12-16]. However, consensus is lacking regarding when the switch should be made. Thus, the objective of the current study was to retrospectively analyze the effect of steroid switch timing on real-life visual and anatomical data in recalcitrant DME eyes with an inadequate response to multiple anti-VEGF injections.

## Materials and methods

This was a retrospective, interventional, comparative study carried out at the Al Bahar Eye Center, a tertiary eye care center in Kuwait City, Kuwait. This real-world study was conducted in accordance with the Declaration of Helsinki and approved by the institutional review board of Al Bahar Eye Center (approval ID: AIRB/021/2020). Informed consent was obtained from all patients included in the study. Chart reviews were performed of the data for consecutively presenting patients with DME at the retina clinic using the available initial retinal imaging and documented treatment failure with anti-VEGF agents (ranibizumab or aflibercept). Treatment failure, following the administration of at least three anti-VEGF injections, was defined as (1) a lack of anatomic improvement (persistent intraretinal cystic changes or macular edema with a central retinal thickness (CRT) of ≥300 microns on spectral domain optical coherence tomography) and (2) a lack of visual improvement (≤1 line of visual gain according to the Snellen acuity chart).

Exclusion criteria were proliferative diabetic retinopathy, tractional retinal detachment, and macular degeneration. Coexisting conditions representing relative or absolute contraindications to the usage of intravitreal dexamethasone implants included ocular or periocular infections (i.e., viral disease of the cornea and conjunctiva, for example, active herpes simplex epithelial keratitis, varicella, mycobacterial infection, and fungal disease). Additional exclusions were aphakic eyes with a ruptured or missing posterior lens capsule and eyes with an anterior chamber intraocular lens. The presence of glaucoma was not an absolute exclusion criterion. Patients with controlled intraocular pressure (IOP) of ≤20 mmHg on anti-glaucoma medication were enrolled in the study. Patients with advanced glaucoma with severe neural rim loss, dense visual field changes, or who required surgical treatment for glaucoma were not included in the study. Patients who did not respond adequately to anti-VEGF treatment were divided into three groups: (1) Group I: patients switched to a steroid implant after three anti-VEGF injections; (2) Group II: patients switched to a steroid implant after four to six anti-VEGF injections; and (3) Group III: patients switched to a steroid implant after more than six anti-VEGF injections.

The primary and secondary endpoints were a change in vision and CRT following the initiation of the implant (i.e., Ozurdex®) and fluctuations in IOP/the identification of adverse events, respectively. The last follow-up took place after 12-16 weeks of Ozurdex® injections. Short, three-month follow-ups were decided upon to rule out the need for repeat injections of the dexamethasone implant or anti-VEGF injections and also the potential decrease in vision due to the development of cataracts.

Standard statistical analysis was performed using MedCalc® statistical software (version 18.5) (MedCalc Software bvba, Ostend, Belgium). Descriptive statistics, mean, standard deviation, and percentages were used, as needed. The paired sample t-test was employed for intragroup comparisons of the normally distributed parameters. The significance level was set at a p-value of <0.050.

## Results

In total, 163 dexamethasone implants were used in recalcitrant DME eyes that had not responded adequately to anti-VEGF injections over the 12-month study period. Fifty-eight of the 163 eyes were excluded from the study for several reasons, including incomplete records and a lack of follow-up. One hundred and five eyes that did not respond adequately to multiple anti-VEGF injections were enrolled in the current study; thereafter, the therapy was switched to a steroid implant. The eyes were divided into three groups according to the number of anti-VEGF injections administered before the switch to the steroid implant. Table 1 shows the demographic profile of all three groups. As can be seen, all groups were statistically matched.

**Table 1:** Demographic profile of the study population VEGF: vascular endothelial growth factor; DM: diabetes mellitus; dex implant: dexamethasone implant

	Group I: Switch after three anti-VEGF injections	Group II: Switch after four to six anti-VEGF injections	Group III: Switch after more than six anti-VEGF injections	P-value
Age (years)	59.41 (4.96) Mean(SD)	59.17 (3.75) Mean(SD)	59.76 (4.68) Mean(SD)	0 .8726
Type I DM (n)	8	5	5	0.936
Type II DM (n)	33	29	25	
Duration of DM (yrs)	16.07 (5.18) Mean(SD)	15.79 (5.31) Mean(SD)	16.73 (4.19) Mean(SD)	0.5793
HbA1C	9.40 (0.96) Mean(SD)	9.39 (1.24) Mean(SD)	9.63 (1.31) Mean(SD)	0.687
Phakic (n)	26	20	20	0.9731
Pseudophakic (n)	15	14	10	
Anti-VEGF used before dex implant switch (aflibercept) (n)	22	17	16	0.9582
Anti-VEGF used before dex implant switch (ranibizumab) (n)	19	17	14	

Table 2 enumerates the details of the three groups in terms of the number of eyes included and the overall vision results. Of the 105 eyes, 41 eyes were switched to a steroid implant after three anti-VEGF injections (Group I); 34 eyes were switched to a steroid implant after four to six injections (Group II), and 30 eyes were switched to a steroid implant after more than six anti-VEGF injections (Group III). In Group I, 19 eyes (46%) showed an improvement in vision after the steroid implant injections; 12 eyes (29%) showed no change in vision; and 10 eyes (24%) experienced a decrease in vision, compared with pre-steroid implant visual acuity. In Group II, 17 eyes (50%) demonstrated an improvement in vision; 15 eyes (44%) experienced the same vision as that at pre-steroid implant levels; and two eyes (6%) showed a decrease in vision. In Group III, 10 eyes (33%) experienced an improvement in vision after the steroid implant injections; eight eyes (27%) did not experience a change in vision; and 12 eyes (40%) showed a decrease in vision, compared to pre-steroid injection levels. In summary, of a total of 105 eyes, 46 eyes (44%) experienced an improvement in vision after the steroid implant injections; 35 eyes (33%) were shown to experience the stabilization of vision (i.e., similar to post-anti-VEGF injection levels); and 24 eyes (23%) experienced a decrease in vision. When analysis was performed of the number of eyes that had improved as well as shown the stabilization of vision after the administration of the dexamethasone implant, it was established that the best visual results were found in Group II, where 94% of eyes showed either an improvement in or the stabilization of vision; comparative figures were 76% of the eyes in Group I and 60% of the eyes in Group III.

**Table 2:** Vision status after dexamethasone switch VEGF: vascular endothelial growth factor; dex implant: dexamethasone implant

Switch	Total number of eyes	Vision improved n (%)	Vision stabilized /same as before dex implant n (%)	Vision worsened n (%)
Group I: Switch after three anti-VEGF injections	41	19 (46.3%)	12 (29.2%)	10 (24.3%)
Group II: Switch after four to six anti-VEGF injections	34	17 (50%)	15 (44.1%)	2 (5.8%)
Group III: Switch after more than six anti-VEGF injections	30	10 (33.3%)	8 (26.6%)	12 (40%)
Total	105	46 (43.8%)	35 (33.3%)	24 (22.8%)

Table 3 reflects the statistical significance associated with the change in vision in all three groups. In Group I, there was a significant improvement in vision after three anti-VEGF injections (p = 0.003). However, since the CRT did not improve as expected (remaining at ≥300 microns), the treating physician decided to switch over to a dexamethasone implant. The vision improved after the dexamethasone implant, but it was not statistically significant (p = 0.143). In Group II, following the administration of four to six anti-VEGF injections, the improvement in vision was without statistical significance (p = 0.638), but after the dexamethasone implant injection, visual improvement was statistically significant (p = 0.007). In Group III, the improvement in vision was statistically significant following the administration of more than six anti-VEGF injections (p = 0.013); however, since the improvement in CRT was unsatisfactory, the treating physician switched the treatment to a dexamethasone implant. After the switch, the eyes in Group III did not maintain the visual gains achieved with the anti-VEGF injections (i.e., a decrease in vision was observed through an increase from the pre-steroid value of 0.703 Logarithm of the Minimum Angle of Resolution (LogMAR) units to 0.756 LogMAR units after the switch). However, this change was without statistical significance (p = 0.229). The last three columns display the baseline vision, the vision at the conclusion of the study period, and the corresponding statistical significance. It is evident that there was an improvement in vision across all three groups, with statistically significant improvements observed in Groups I and II.

**Table 3:** Statistical changes in vision status after dexamethasone switch VEGF: vascular endothelial growth factor; dex implant: dexamethasone implant

Group	Baseline vision (mean)	Vision after anti-VEGF injections (mean)	P-value	Vision after anti-VEGF injections (Mean)	Final vision after dex implants (mean)	P-value	Baseline vision (mean)	Final vision after dex implants (mean)	P-value
Group I: Switch after three anti-VEGF injections	0.738	0.607	0.003	0.607	0.534	0.143	0.738	0.534	0.001
Group II: Switch after four to six anti-VEGF injections	0.651	0.631	0.638	0.631	0.555	0.007	0.651	0.555	0.012
Group III: Switch after more than six anti-VEGF injections	0.885	0.703	0.013	0.703	0.756	0.229	0.885	0.756	0.046

Table 4 shows the change in CRT before and after the steroid switch in our study population. The majority of eyes in the current study showed a decrease in CRT following the steroid implant injections. In Group I, of 41 eyes, 38 eyes (93%) showed a decrease in CRT, and thicker retinas were seen in three eyes (7%), compared to pre-steroid levels. In Group II, 29 eyes (85%) experienced a decrease in CRT, and five eyes (15%) experienced an increase in CRT, compared to pre-steroid levels. In Group III, 26 eyes (87%) reflected a decrease in CRT, and four eyes (13%) were demonstrated to have increased CRT compared to pre-steroid injection levels. Thus, overall, of 105 eyes, 93 (89%) experienced a decrease in CRT; however, an absolute improvement in vision was only seen in 46 (44%) eyes following the dexamethasone implant injections.

**Table 4:** Central retinal thickness (CRT) after dexamethasone switch VEGF: vascular endothelial growth factor; dex implant: dexamethasone implant; OCT: optical coherence tomography

Switch	Number of eyes	Mean CRT before dex implant mean (SD)	Mean CRT after dex implant mean (SD)	p-value
Group I: Switch after three anti-VEGF injections	41	482.5 (+/- 144.7)	331.6 (+/- 127.2); OCT decrease in 38 eyes (93%)	0.00001
Group II: Switch after four to six anti-VEGF injections	34	444.0 (+/- 95.87)	359.02 (+/-117.8); OCT decrease in 29 eyes (85%)	0.001
Group III: Switch after more than six anti-VEGF injections	30	469.26 (+/- 88.6)	387.6 (+/-144.4); OCT decrease in 26 eyes (87%)	0.010

The effects of steroid implant injections on IOP were assessed in the three groups. Of the 105 eyes, 98 did not have a prior history of glaucoma (non-glaucoma group). Seven eyes were known to have glaucoma, but they were stabilized with anti-glaucoma medications.

Table 5 depicts the IOP fluctuations during the study period. In the non-glaucoma group, 12 eyes (12%) reported an IOP of ≥21 mmHg. The highest spike was 27 mmHg, and, on average, spikes were seen two weeks post steroid injections. Anti-glaucoma medication was not started, and the IOP returned to pre-injection levels in all eyes. In cases with a known history of glaucoma, an IOP of ≥21 mmHg was observed (n = 3 eyes, 43%). New anti-glaucoma medication was not started in these cases. All the eyes responded well to the existing glaucoma medications. Surgical intervention was not warranted to control IOP in any of the patients.

**Table 5:** Intraocular pressure (IOP) changes with dexamethasone implant

Group	Number of eyes	Initial IOP mean (+/- SD)	Day 1 mean (+/- SD)	Week 2 mean (+/- SD)	Month 1 mean (+/- SD)	Month 2 mean (+/- SD)	Month 3 mean (+/- SD)
No glaucoma	98	15.46 (1.37)	16.06 (1.91)	16.22 (2.24)	16.88 (1.88)	15.91 (1.73)	16.16 (1.49)
Known glaucoma	7	17.39 (1.28)	17.57 (1.46)	20.85 (5.8)	20.14 (1.34)	18.28 (0.75)	17.71 (1.11)

In the current study, 37 eyes (35%) were pseudophakic, and 68 eyes (65%) were associated with various grades of cataracts. The study was planned with a short follow-up duration of three months, intentionally, to avoid the bias linked to the increase in cataracts during the vision assessment.

## Discussion

The management of DME refractory to anti-VEGF injections is challenging, and the treatment options are limited. The efficacy of steroid implants in refractory, persistent DME eyes initially treated with other modalities (laser, intravitreal anti-VEGF, and triamcinolone acetonide (TA)) has been reported in several studies [12-17]. In the current study, DME eyes resistant to anti-VEGF therapy were switched over to dexamethasone implants at the discretion of the treating physician. The main requisite for enrollment in the study was that at least three anti-VEGF injections had to have been performed prior to switching to a steroid implant.

When the findings for all three groups were compared, an interesting finding was that, overall, the best vision results (from baseline to the final follow-up) were seen in Group I; however, when the improvement in vision was recorded after the switch to steroids, optimal results were observed in Group II (i.e., the switch made after four to six anti-VEGF injections). The current study was not powered to determine the reason for better vision results in Group II compared to the other groups; however, it was hypothesized that this may have been owing to the ceiling effect of visual improvement in Group I. After three anti-VEGF injections, robust visual improvement occurred, but either the visual or the structural improvement identified on optical coherence tomography (OCT) was not satisfactory enough. Hence, the treating clinician decided to switch to a dexamethasone implant. Although the best overall vision was attributed to Group I, the finding of an improvement in vision after the switch to a steroid was not statistically significant. By contrast, statistically significant visual improvements were identified in Group II after the dexamethasone implant injections (i.e., when the switch to a steroid was made after four to six anti-VEGF injections). The longer DME duration (i.e., the switch to a steroid after more than six anti-VEFG injections) elucidated why the improvement in vision occurred to a smaller extent in Group III. Refractory DME eyes are associated with an inferior functional outcome compared to naïve eyes, owing to longstanding edema, which can cause structural damage to the outer retinal layers. Prolonged underlying capillary ischemia, if present, explains the increase in photoreceptor damage in chronic or refractory DME eyes. In the current study, the ischemic changes were not evaluated. Interestingly, when persistent DME eyes were switched to a steroid implant, the improvement in vision achieved by the anti-VEGF injections, albeit without statistical significance, was not sustainable in several eyes.

In Group I, 10 eyes (24%) showed a decrease in vision, whereas two eyes (6%) and 12 eyes (40%) in Groups II and III, respectively, experienced a decrease in vision after the steroid implant injections. In Group II, where the switch to a steroid was performed after four to six anti-VEGF injections, the least number of eyes experienced a decrease in vision after the anti-VEGF injections. The current retrospective study included a small sample; thus, it was not powered to determine the cause of the decrease in vision. It was hypothesized that the eyes in Group I achieved satisfactory results with the anti-VEGF injections (i.e., an improvement in vision that was statistically significant) (p = 0.003). Presumably, while on anti-VEGF therapy, they could have continued to negate the VEGF load, and the switch to a steroid implant would have been made at a later stage to address the inflammatory component of DME pathophysiology. This hypothesis could neither be proved nor verified because there was no control group.

In a similar study performed by Medrano et al. [12], 108 previously treated eyes with inadequate responses to anti-VEGF therapy were allocated to two groups according to the number of injections administered. Groups I and II received three and more than three anti-VEGF injections, respectively. The authors reported that at month 12, best corrected visual acuity significantly improved from 0.31 ± 0.22 at baseline to 0.37 ± 0.23 (p = 0.006). The CRT in previously treated eyes reduced significantly from 431.3 ± 115.5 µm at baseline to 269.3 ± 66.2 µm at month 12 (p = < 0.000). The authors concluded that eyes with an insufficient response to anti-VEGF, switching from dexamethasone at the time to three-monthly anti-VEGF inhibitor injections, provided better functional outcomes than those that received more than three anti-VEGF injections. These findings are also reflected in our study data. In our study, Group III patients who were switched to the dexamethasone implant late, after more than six anti-VEGF injections, showed poor visual results when compared to Group I and II patients who were switched to the dexamethasone implant earlier after the anti-VEGF trial.

In another retrospective, single-center study carried out by Martinez et al. [13], 31 of 69 eyes received three anti-VEGF injections prior to an early switch to a steroid, and the remaining 38 eyes received more than six anti-VEGF injections prior to a late switch to a steroid. In the early-switch group, best corrected visual acuity increased significantly from 0.2 at baseline to 0.4 at month 24 (p = 0.004). By contrast, in the late-switch group, best corrected visual acuity did not increase (p = 0.860). Central subfoveal thickness was significantly reduced in both the early- and late-switch groups (p = 0.000 and p = 0.004, respectively). In the current study, of 105 eyes, 93 (89%) showed a decrease in CRT and visual improvement/stabilization in 81 eyes (77%) following dexamethasone implant injections. After analysis, the best vision-based results were observed in Group II, where 94% of the eyes showed either an improvement in or the stabilization of vision, compared to 76% of eyes in Group I and 60% of eyes in Group III. Visual improvement was observed in all three groups following the switch to dexamethasone implants compared to baseline vision. Notably, Groups I and II, which underwent the dexamethasone implant switch earlier in their treatment, experienced greater vision improvement than Group III, where the switch occurred after more than six anti-VEGF injections. Interestingly, Group III did not maintain the visual gains achieved with the anti-VEGF injections, as evidenced by a decrease in vision from 0.703 LogMAR units to 0.756 LogMAR units after the dexamethasone implant switch. However, this change was not statistically significant.

The most common concern about the intraocular use of corticosteroids is the increase in IOP. However, in some studies, the elevation in IOP appeared to be lower in dexamethasone implant patients compared to those treated with a fluocinolone acetonide implant and triamcinolone acetonide [17,18]. In a 12-month BEVORDEX trial, eyes with IOP >25 mmHg at least once during the follow-ups were dexamethasone-implanted eyes (26%) versus none in the bevacizumab group [18]. Those with increased IOP were managed with observation or topical IOP-lowering medications. In the three-year MEAD study, the percentage of IOP of ≥25 mmHg at any follow-up was 32%, 27%, and 4% in the 0.70 mg and 0.35 mg DEX implant and sham groups, respectively; one patient in the 0.70 mg and 0.35 mg DEX implant group required a trabeculectomy [19]. In the current study, 15 eyes (14%) were demonstrated to have an IOP >21 mmHg after the steroid implant injections. A new anti-glaucoma medication was not started in these eyes, nor was surgical intervention necessary to control IOP in any of the patients in the current study.

The development or progression of cataracts in phakic patients is a side effect of corticosteroid treatment. Cataract progression cannot be assessed in studies with a follow-up period of less than six months. A three-month follow-up period was purposefully chosen in the current study to avoid the bias associated with an increase in cataracts during the vision assessment. Furthermore, later in the course of their treatment, the eyes enrolled in our study required a variety of treatment modalities, such as laser, anti-VEGF, and repeat dexamethasone implants. The primary goal of our study was to determine the absolute functional and morphological effect of dexamethasone implant switch timing on anti-VEGF-resistant DME eyes. If we had extended our follow-up period, we would have received mixed treatment data and the results of combination therapy. This study has limitations, one of which is its retrospective design. With a case series, as with any retrospective study, the results and conclusion depend on the availability and accuracy of the medical records. Secondly, the cases were self-selected, and there was no control group. In addition, it was a single-center study with a limited number of patients. Finally, the follow-up period was short; hence, caution needs to be employed when drawing conclusions.

## Conclusions

The use of intravitreal dexamethasone implants showed both morphological and visual enhancements in eyes with anti-VEGF-resistant DME. In our study, vision improved in 46 eyes (44%), and morphologically, 93 eyes (89%) showed a decrease in CRT. When comparing the visual outcomes of three distinct groups, it was observed that Group I, which underwent a switch to steroids after three anti-VEGF injections, exhibited the most substantial improvement in vision from baseline to the final follow-up. Notably, the majority of visual improvement in this group occurred following the anti-VEGF loading dose before the administration of steroids. An intriguing finding emerged when analyzing the vision improvement subsequent to the switch to steroids in Group II, particularly among those who transitioned after receiving four to six anti-VEGF injections, as they demonstrated the most optimal results. While Group I achieved the best overall vision, the improvement in vision after the switch to steroids was not statistically significant. Conversely, statistically significant visual enhancements were noted in Group II following the dexamethasone implant injections, specifically when the switch to steroids occurred after four to six anti-VEGF injections. Overall, the results suggest that the timing of the switch to dexamethasone implants in eyes with anti-VEGF-resistant DME may play a crucial role in achieving optimal visual outcomes.
